# Supplementary material for: Rapid Loss of CD4 T Cells by Pyroptosis during Acute SIV Infection in Rhesus Macaques
Source: J Virol. 2022 Aug 24;96(17):e00808-22. doi: 10.1128/jvi.00808-22 (PMC9472632; doi:10.1128/jvi.00808-22)
Supplement: Supplemental file 1 — Fig. S1 to S8. Download jvi.00808-22-s0001.pdf, PDF file, 4.5 MB [file jvi.00808-22-s0001.pdf]

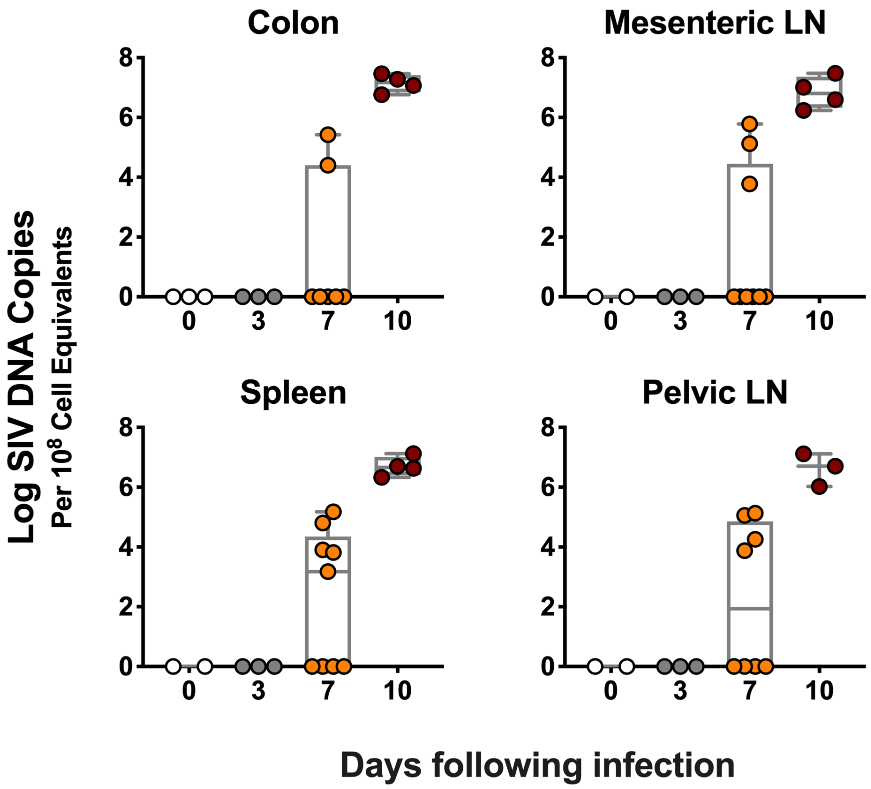

Figure S1. Dynamics of viral DNA in tissues. Viral DNA (log RNA copies/10^8 cell equivalents) in multiple tissues at necropsies in monkeys on days 0, 3, 7 and 10 following SIVmac251 infection.

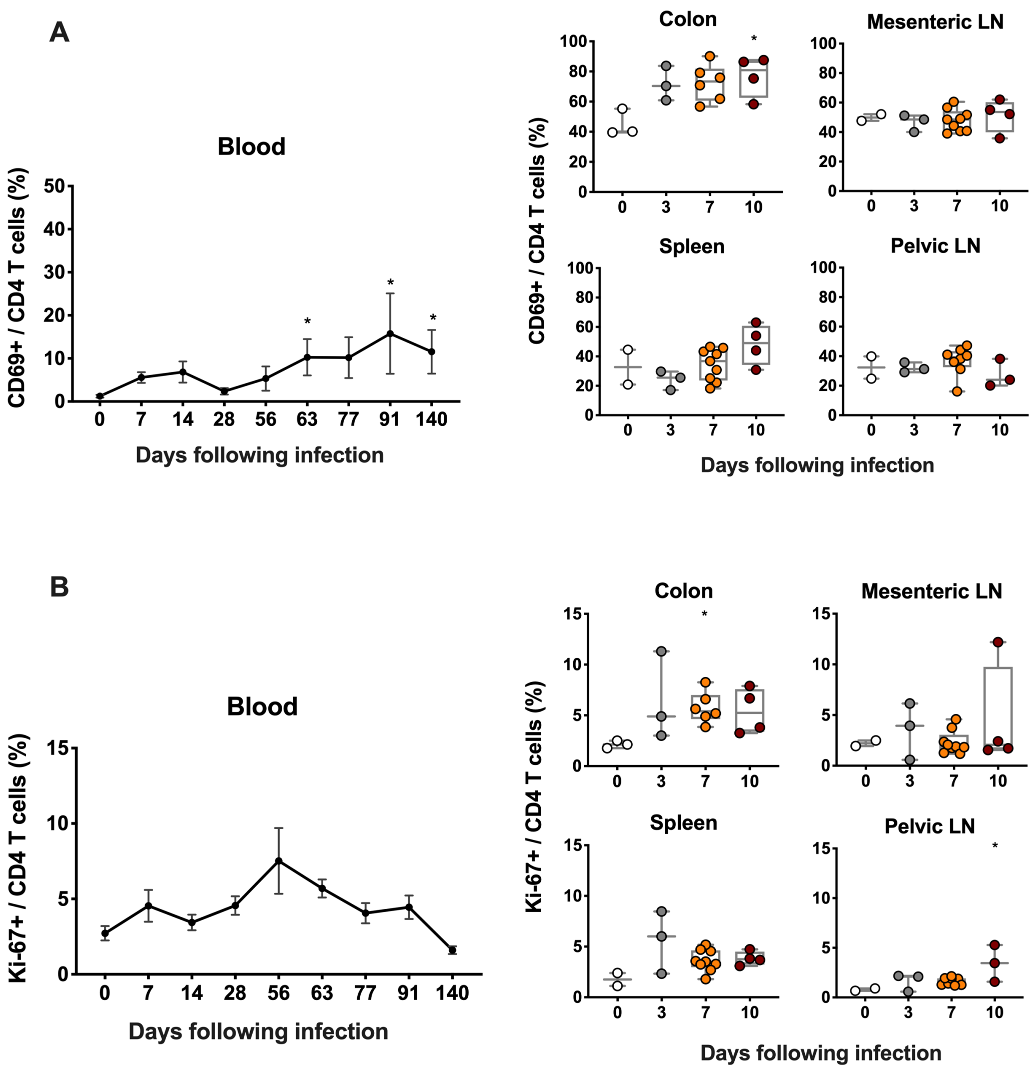

Figure S2. The expression of CD69 and Ki67 in CD4 T cells from blood and tissues. (**A**) CD69 expression of CD4 T cells from blood and multiple tissues at necropsy during the course of SIVmac251 infection. (**B**) Ki67 expression of CD4 T cells in blood and multiple tissues at necropsy in monkeys with SIVmac251 infection. Significant *p* values are shown in figures. Experimental variables were analyzed by one-way analysis of variance (ANOVA). * *p .05*

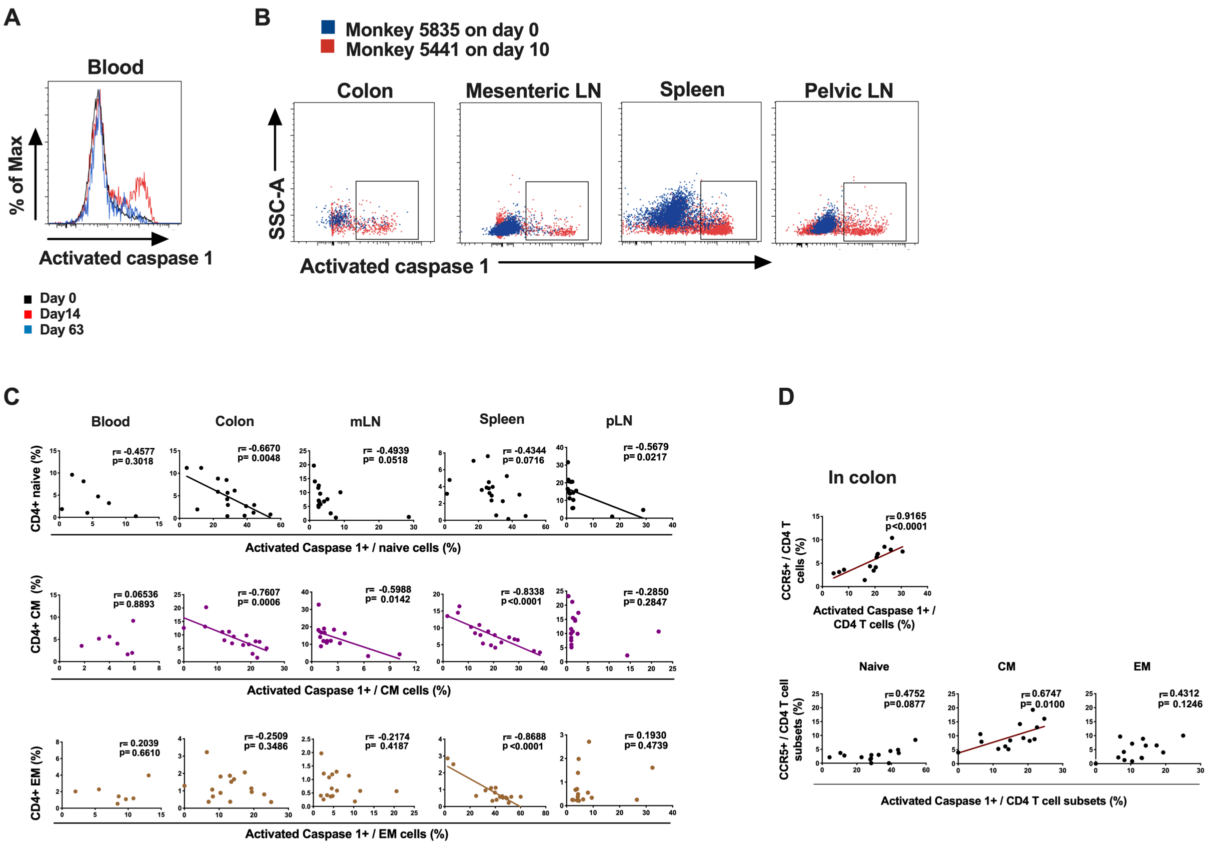

Figure S3. Detection of CD4 T cell pyroptosis during SIV infection. (**A**) Representative flow cytometric histogram indicating pyroptosis of CD4 T cells in blood from one monkey on days 0, 14 and 63. (**B**) Representative flow cytometric plots showing CD4 T cells expressing activated caspase 1 in multiple tissues from two monkeys with or without SIV infection. (**C**) Correlation between the expression of activated caspase 1 in CD4 T cell subsets including naive, central memory (CM), effector memory (EM) subsets and their corresponding level in blood and tissues. (**D**) Correlation between CCR5 expression in CD4 T cells or CD4 T cell subsets and expression of activated caspase 1 in CD4 T cells or CD4 T cell subsets in colon. Solid lines indicate the correlation with significant p values. spearman rank order correlation coefficients r and corresponding p values are indicated.

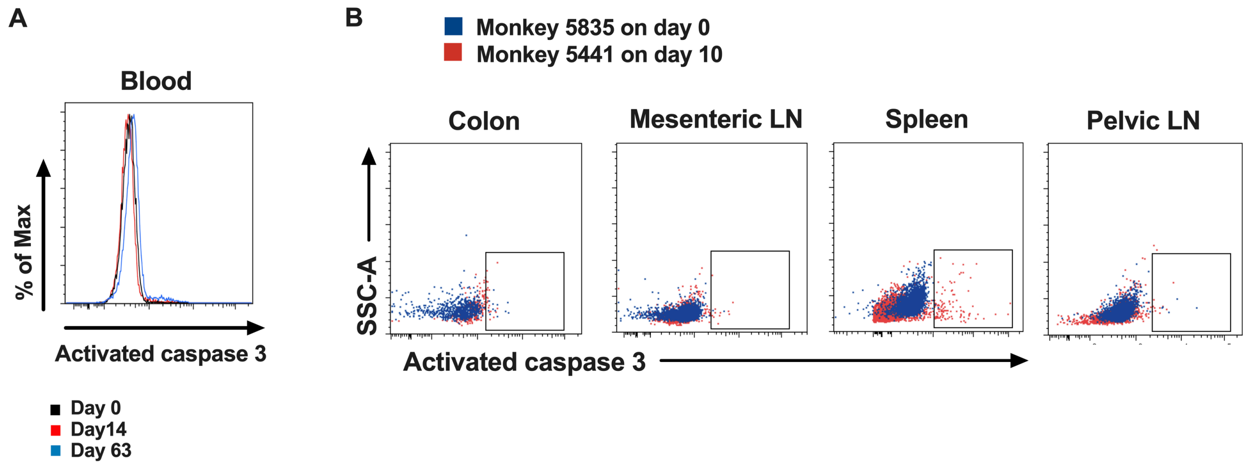

Figure S4. Detection of CD4 T cell apoptosis during SIV infection. (**A**) Representative flow cytometric histogram indicating apoptosis of CD4 T cells in blood from one monkey on days 0, 14 and 63. (**B**) Representative flow cytometric plots showing CD4 T cells expressing activated caspase 3 in multiple tissues from two monkeys with or without SIV infection.

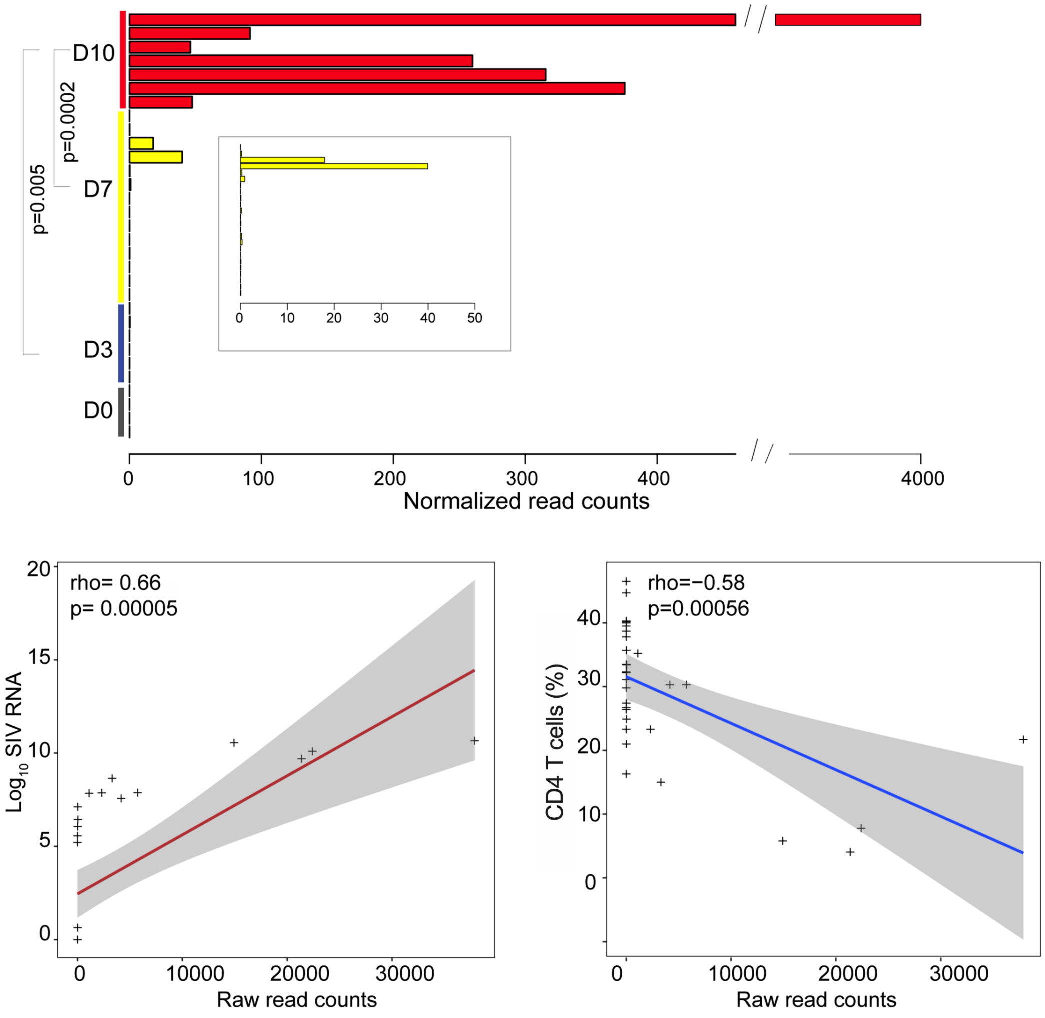

Figure S5. Distribution of RNA-Seq reads that were mapped to the SIV genome at days 0, 3, 7 and 10. At the top, reads that did not map the rhesus macaque genome were mapped to the SIV genome. Counts per millions reads was plotted for each sample at each time point. Enrichment of SIV mapped reads at day 10 compared to day 3 and day 7 was assessed using Wilcox rank test. At the bottom, spearman correlation between the number of reads mapped to the SIV genome for each animal and SIV RNA or the frequency of CD4 T cells. Each dot on the plot represents an animal. Gray are representing the plot interval confidence (IC) of 95%.

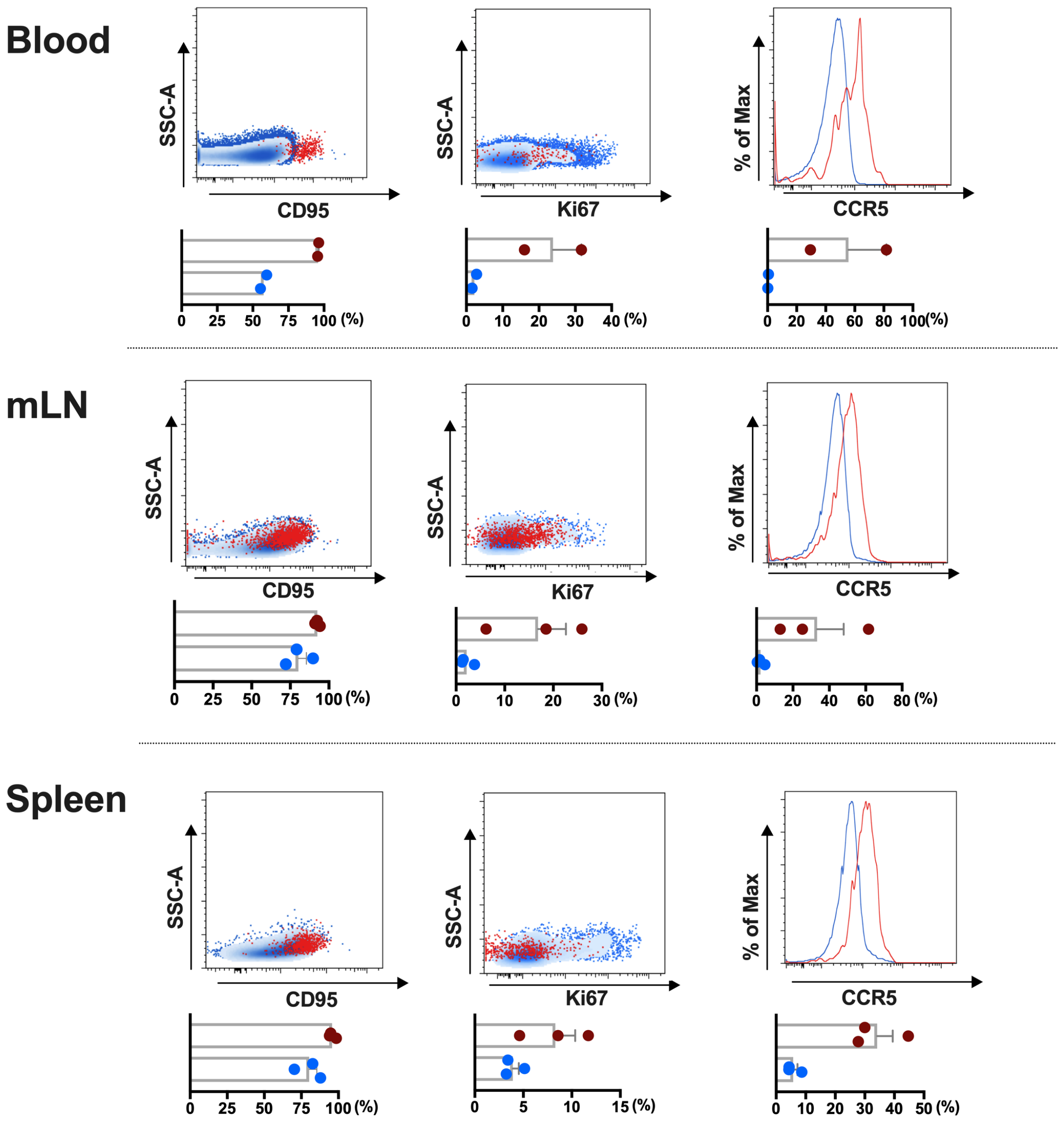

Figure S6. Characterization of CD4 T cells with translation-competent SIV in blood, mesenteric LNs and spleen from SIV-infected monkeys during early SIV infection. From left to right, representative flow cytometric plots or histogram showing CD4 T cells expression CD95, ki67 and CCR5, respectively. Uninfected CD4 T cells are shown in blue, SIV+CD4 T cells are shown in red. The corresponding data are also summarized in columns below.

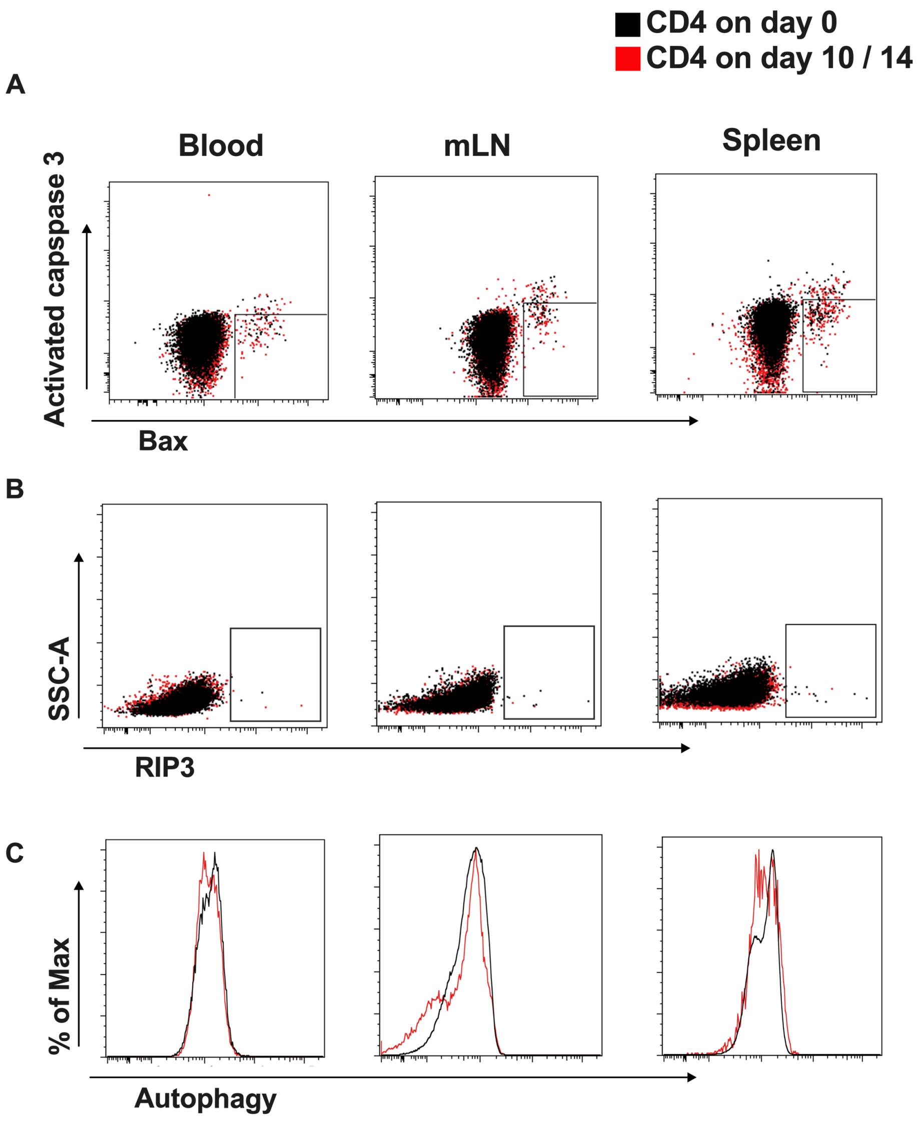

Figure S7. CD4 T cells capable of undergoing mitochondria-induced caspase-independent cell death, necroptosis or autophagy in blood and tissues. (**A**) Representative flow cytometric plots showing CD4 T cells containing Bax but without caspase 3 expression in blood and tissues from naive (black) or infected (red) monkeys. (**B**) Representative flow cytometric plots showing CD4 T cells expressing RIP3 in blood and tissues from naive (black) or infected (red) monkeys. (**C**) Representative flow cytometric plots showing CD4 T cells staining with autophagy probes in blood and tissues from naïve (black) or infected (red) monkeys.

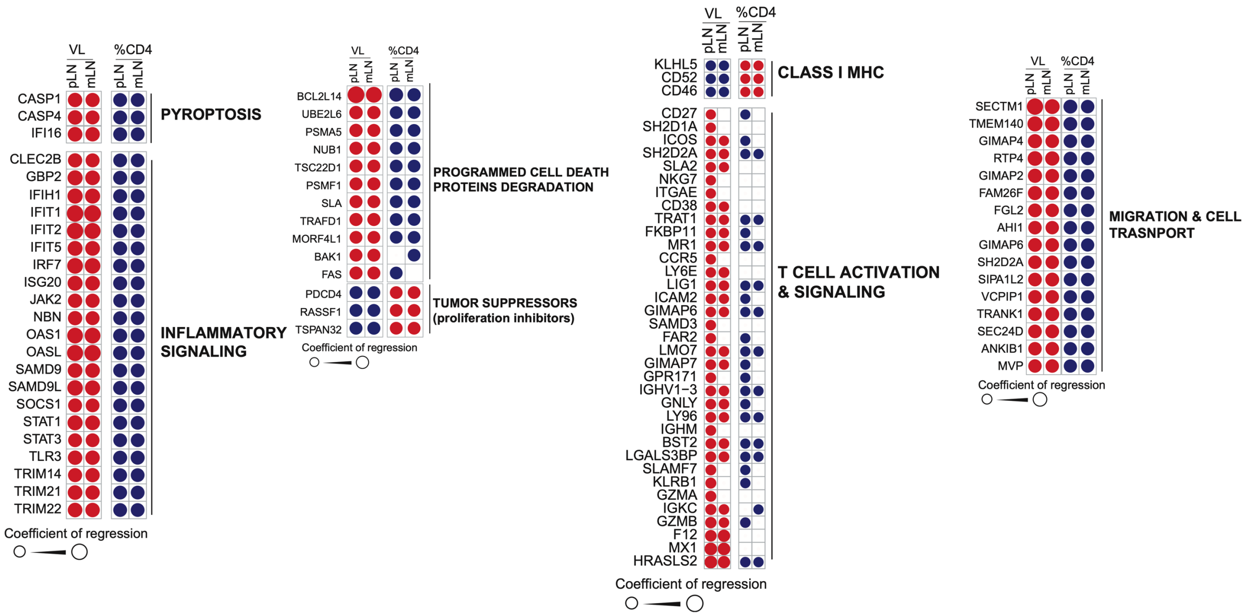

Figure S8. Markers of pyroptosis, inflammatory response, programmed cell death and protein degradation, tumor suppressors, class MHC I, T cell activation and signaling, cell migration and transport that correlated significantly with viral RNA and the frequency of CD4 T cells in pLN and mLN. Red and blue circles show the regression coefficient of the expression of each marker significantly correlated with viral RNA or the frequency of CD4 T cells, respectively ($p < 0.05$). Red stands for positive correlation and blue stands for negative correlation. The size of the circle represents the level of the correlation where bigger the size of the circle, higher is the correlation of this gene with viral RNA or frequency of CD4 T cells.
